# Common genetic variation in IGF1, IGFBP-1, and IGFBP-3 in relation to mammographic density: a cross-sectional study

**DOI:** 10.1186/bcr1655

**Published:** 2007-02-14

**Authors:** Rulla M Tamimi, David G Cox, Peter Kraft, Michael N Pollak, Christopher A Haiman, Iona Cheng, Matthew L Freedman, Susan E Hankinson, David J Hunter, Graham A Colditz

**Affiliations:** 1 Channing Laboratory, Department of Medicine, Brigham and Women's Hospital and Harvard Medical School, 181 Longwood Avenue, Boston, MA 02115, USA; 2 Department of Epidemiology, Harvard School of Public Health, 677 Huntington Avenue, Boston, MA 02115, USA; 3 Department of Biostatics, Harvard School of Public Health, 677 Huntington Avenue, Boston, MA 02115, USA; 4 Department of Medicine and Oncology, McGill University, 3755 Cote Ste-Catherine Road, Montreal, Quebec H3T 1E2, Canada; 5 Keck School of Medicine, University of Southern California, 1441 Eastlake Avenue, Los Angeles, CA 90089, USA; 6 Department of Epidemiology and Biostatistics, University of California, 513 Parnassus Avenue S966B, San Francisco, CA 94143, USA; 7 Program in Medical and Population Genetics, Broad Institute of Massachusetts Institute of Technology and Harvard, 7 Cambridge Center, Cambridge MA 02142, USA; 8 Department of Surgery, Washington University School of Medicine, Campus Box 8100 660, S. Euclid Avenue, St. Louis, MO 63110 USA

## Abstract

**Introduction:**

Mammographic density is one of the strongest risk factors for breast cancer and is believed to represent epithelial and stromal proliferation. Because of the high heritability of breast density, and the role of the insulin-like growth factor (IGF) pathway in cellular proliferation and breast development, we examined the association between common genetic variation in this pathway and mammographic density.

**Methods:**

We conducted a cross-sectional analysis among controls (*n *= 1,121) who were between the ages of 42 and 78 years at mammography, from a breast cancer case-control study nested within the Nurses' Health Study cohort. At the time of mammography, 204 women were premenopausal and 917 were postmenopausal. We genotyped 29 haplotype-tagging SNPs demonstrated to capture common genetic variation in IGF1, IGF binding protein (IGFBP)-1, and IGFBP-3.

**Results:**

Common haplotype patterns in three of the four haplotype blocks spanning the gene encoding IGF1 were associated with mammographic density. Haplotype patterns in block 1 (*p *= 0.03), block 3 (*p *= 0.009), and block 4 (*p *= 0.007) were associated with mammographic density, whereas those in block 2 were not. None of the common haplotypes in the three haplotype blocks spanning the genes encoding IGFBP-1/IGFBP-3 were significantly associated with mammographic density. Two haplotype-tagging SNPs in IGF1, rs1520220 and rs2946834, showed a strong association with mammographic density. Those with the homozygous variant genotype for rs1520220 had a mean percentage mammographic density of 19.6% compared with those with the homozygous wild-type genotype, who had a mean percentage mammographic density of 27.9% (*p *for trend < 0.0001). Those that were homozygous variant for rs2946834 had a mean percentage mammographic density of 23.2% compared with those who were homozygous wild-type with a mean percentage mammographic density of 28.2% (*p *for trend = 0.0004). Permutation testing demonstrated that results as strong as these are unlikely to occur by chance (*p *= 0.0005).

**Conclusion:**

Common genetic variation in IGF1 is strongly associated with percentage mammographic density.

## Introduction

Mammographic density is one of the strongest risk factors for breast cancer. Women with 75% or more breast density are at a fourfold to sixfold greater risk of breast cancer than women with no density [1,2]. It has been hypothesized that mammographic density reflects cumulative exposure to estrogens [3]; however, accumulating evidence suggests that the mechanism by which mammographic density influences breast cancer may be independent of circulating estrogen levels [4-6].

The insulin-like growth factor (IGF) pathway has a critical role in cell proliferation, as well as in the growth and development of the breast [7]. Because mammographic density is associated with epithelial and stromal proliferation [8,9], and circulating IGF1 and IGF binding protein (IGFBP)-3 are associated with premenopausal breast cancer in some studies [10,11], but not all [12-14], the IGF pathway is a compelling candidate for examination with respect to mammographic density. Circulating IGF1 levels have been positively associated with mammographic density [15,16], and IGFBP-3 levels inversely with mammographic density in premenopausal women [15-17], although no association has been observed in postmenopausal women [15,16].

It is estimated from twin studies that genetics accounts for 60 to 67% of the variation in mammographic density [18]. Given the high degree of heritability [18,19], identifying the genes involved is important for understanding the biology of breast density and how it influences breast cancer risk. Several studies have addressed the role of polymorphisms in estrogen synthesis and metabolizing genes and mammographic density, with inconclusive results [20-26]. Evidence relating the -202 promoter SNP (rs2854744) in IGFBP-3 to mammographic density has also been mixed [27,28]. No studies so far have examined the association between polymorphisms in IGF1 or IGFBP-1 and mammographic density. Genetic variation in genes involved in the IGF pathway may reflect long-term or lifetime exposure of circulating levels of IGF1, IGFBP-1, and IGFBP-3. We conducted a cross-sectional study in the Nurses' Health Study (*n *= 1,121) to assess the relation between common genetic variation in these genes and mammographic density. So far, no study has comprehensively examined the relation between common genetic variation in these genes and mammographic density.

## Materials and methods

### Study design and population

The Nurses' Health Study was initiated in 1976, when 121,700 US registered nurses aged 30 to 55 years returned an initial questionnaire [29]. Information on body mass index (BMI), reproductive history, age at menopause, and postmenopausal hormone use as well as diagnosis of cancer and other diseases are updated every 2 years through questionnaires. During 1989 and 1990, blood samples were collected from 32,826 women. Detailed information regarding blood collection methods has been published [30]. In general, blood samples were returned within 26 hours of blood draw, then immediately centrifuged, separated into plasma, red blood cells, and buffy coat fractions, and stored in freezers under liquid nitrogen. The follow-up rate among women who provided blood samples was 99% up to and including 1998.

We conducted a cross-sectional analysis among controls from a breast cancer case-control study nested within the Nurses' Health Study cohort. This nested case-control study, examining plasma markers and genetic variation with respect to breast cancer risk, included breast cancer cases diagnosed after blood collection but before 1 June 1998, and matched controls [31]. Controls were matched to cases on year of birth, menopausal status, postmenopausal hormone use, time of day, month, and fasting status at time of blood draw. Mammography collection was targeted to 1,329 breast cancer controls, with DNA samples, through the 1998 follow-up cycle. At the time of mammography collection, 1,297 of these participants were alive and eligible to receive letters for participation in this study. Of these women, 1,189 (92%) gave permission to obtain mammograms; 5% did not give permission, and 3% reported not having had a mammogram. For all consenting women, we attempted to obtain the mammograms taken as close to the date of blood collection as possible. We successfully obtained mammograms from 1,142 controls (96% of those consenting); 21 of these were excluded for not having usable film mammograms. Women for whom we obtained usable mammograms (*n *= 1,121) were very similar to those whom we were unable to obtain mammograms with respect to age, BMI, and circulating hormone levels [4]. This study was approved by the Committee on the Use of Human Subjects in Research at Brigham and Women's Hospital.

### Mammographic density measurements

To assess mammographic density, the craniocaudal views of both breasts were digitized at 261 μm per pixel with a Lumysis 85 laser film scanner, which covers a range of 0 to 4.0 optical density. The software for computer-assisted thresholding was developed at the University of Toronto [32]. The film screen images were digitized and viewed on the computer screen. For each image, the observer set one threshold level to define the edge of the breast and a second threshold delineating the dense area of the breast, within the original threshold region. The Cumulus software calculated the total number of pixels within the entire region of interest and within the area identified as dense. Using these values, the software program calculated the percentage of the breast area that was dense. This measure of mammographic breast density was highly reproducible within this study. The within-person intraclass correlation coefficient was 0.93 [33]. We used the average percentage density of both breasts for this analysis. Previous studies have shown similar results when the breast density of a random side (right or left) or the average of the two are used [16]. We also evaluated the association of IGF1, IGFBP-1, and IGFBP-3 genotypes and haplotypes with the absolute area of mammographic density, but because results were similar and percentage breast density has consistently been a stronger predictor of breast cancer risk, we present the results for percentage mammographic density only.

### SNP selection and genotyping

SNP discovery and haplotype-tagging SNP selection was conducted in the Multiethnic Cohort [34,35]. Novel SNPs were identified by resequencing of the exons in IGF1, IGFBP-1, and IGFBP-3 in 95 cases of advanced prostate cancer and 95 advanced breast cancer from equal numbers of US Caucasians, Latinos, Japanese, native Hawaiians, and African Americans [36].

To identify regions of strong linkage disequilibrium, 64 SNPs in the gene encoding IGF1 and 36 SNPs in the genes encoding IGFBP-1 and IGFBP-3 were genotyped in a panel of 349 cancer-free women from the Multiethnic Cohort [37,38]. Pairwise linkage disequilibrium between SNPs was determined with the *D' *statistic [39]. Regions of strong linkage disequilibrium (that is, haplotype blocks) were defined with the use of criteria from Gabriel and colleagues [40]. Haplotype-tagging SNPs were selected for a Caucasian population using the program TagSNPs [41]. In brief, the selection of haplotype-tagging SNPs is based on *R*^2^_H_, a measure of the correlation between observed haplotypes and those predicted by the tagging SNP genotypes [42].

Fourteen SNPs tag the common haplotypes in four haplotype blocks of strong linkage disequilibrium in the gene encoding IGF1, and 13 SNPs tag the common haplotype patterns in three haplotype blocks across the genes encoding IGFBP-1 and IGFBP-3 among Caucasians (Table 1). Two additional SNPs (rs6670 and rs2453839) in IGFBP-3 did not fall into these blocks. As part of the Breast and Prostate Cancer Cohort Consortium (BPC3), these 29 SNPs were genotyped in the controls from the breast cancer nested case-control study described above (Table 1).

### Genotyping

Genotyping was conducted with a fluorescent 5' endonuclease assay and the ABI-PRISM 7900 for sequence detection (TaqMan) [37,38]. Assay characteristics for genotyping the haplotype-tagging SNPs in IGF-1, IGFBP-1, and IGFBP-3 are available on a public website [43]. For quality control, approximately 10% of samples were included as blinded duplicates in the genotyping runs. The concordance for replicate samples was greater than 99%.

### Statistical analysis

Haplotype frequencies were estimated by using expectation substitution [44,45] implemented in SAS PROC haplotype (SAS Institute, Cary, NC, USA). Haplotypes occurring at frequencies less than 0.05 were grouped together as a composite category.

We used haplotype–trend regression models to determine whether haplotypes are associated with mammographic density [44,45]. Because percentage mammographic density is not normally distributed, we used a square-root transformation to improve the normality of the distribution. Thus, square-root-transformed percentage mammographic density was the dependent variable in multivariate linear regression models with individual haplotypes as independent variables. To determine percentage mammographic density for women with specific genotypes, we back-transformed assuming the covariates of typical woman in this study (namely postmenopausal, 57 years old, BMI of 25, no family history of breast cancer, no personal history of benign breast disease, and so on). We assumed an additive model in which haplotype-specific β parameters represent the per-haplotype increase in square-root-transformed percentage mammographic density. To test the global null hypothesis of no association between the common haplotype patterns within each haplotype block and percentage mammographic density, we computed *F *statistics comparing the model with covariates and block-specific haplotypes with a model without haplotypes. To assess whether specific haplotypes in each block were associated with mammographic density, we included each haplotype in the model individually in comparison with all other haplotypes. Rare haplotypes, with a combined frequency of less than 5%, were not included in analyses.

To determine whether mammographic density is associated with single causal variants, we also examined the relation between the individual haplotype-tagging SNPs in IGF1, IGFBP-1, and IGFBP-3 in relation to mammographic density. We used generalized linear models adjusted for covariates to determine the mean percentage breast density according to genotype. To determine whether there was a linear trend with increasing variant alleles, we calculated *P *values from Wald statistics including an ordinal variable for genotype.

Covariate information at the time of the mammogram was assessed by using data from biennial questionnaires before the date of the mammogram. We included the following known predictors of mammographic density in multivariate models: age (continuous), BMI (continuous), alcohol consumption (none, less than 5 g/day, 5 to 14.9 g/day, more than 15 g/day), age at first birth/parity (nulliparous, age at first birth less than 25 years, age at first birth 25 to 29 years, age at first birth 30 years or more), history of benign breast disease (yes/no), family history of breast cancer (yes/no), menopausal status/postmenopausal hormone use (premenopausal, postmenopausal never user, postmenopausal current user, postmenopausal past user). Although it is unlikely that these factors are confounders of the haplotype and mammographic density relationship, these variables do explain substantial variation in the outcome. Estimates of β were very similar between age-adjusted and multivariate-adjusted models; we therefore present the multivariate-adjusted results only.

Because of the multiple testing in these analyses, we conducted permutation testing to assist in interpreting statistically significant associations [46,47]. Mammographic density was randomly permuted 10,000 times, and all haplotype-tagging SNPs and haplotypes in IGF1 were tested for association with the permuted outcome. The smallest observed *p *value over these tests was compared with the distribution of permuted *p *values. For example, if the lowest observed *p *value was 0.05 and this value marked the 25th centile of the permuted distribution, then the permutation *p *value would be 0.25.

Percentage mammographic density is lower in postmenopausal women than in premenopausal women. However, because the estimates of β were very similar between both groups of women, we present the results for all women combined, taking menopausal status into account in the analysis. Data analysis was conducted with SAS statistical software version 9.1 (SAS Institute, Cary, NC, USA). All *p *values presented are two-sided tests of statistical significance.

## Results

This cross-sectional study examining common genetic variation in the IGF pathway and mammographic density included 1,121 women between the ages of 42 and 78 years at mammography. A total of 204 women were premenopausal at the time of their mammogram, with a mean age at mammography of 49.1 ± 3.4 years (results are shown as means ± SD). Among premenopausal women, the mean percentage mammographic density was 39.5 ± 22.4 (range 0.6 to 90.3). In all, 917 women were postmenopausal at the time of their mammogram, with a mean age at mammography of 60.7 ± 6.1 years. Among postmenopausal women, the mean percentage mammographic density was 23.7 ± 18.8 (range 0.0 to 86.5). Of the postmenopausal women, 34% were currently using postmenopausal hormones, and 22.7% were former users at the time of their mammograms. Of the women in the study, 98.8% were Caucasian. Women who provided a blood sample in the Nurses' Health Study are similar to non-Hispanic white women in SEER (Surveillance Epidemiology and End Results) with regard to breast cancer risk [48,49].

Using haplotype-tagging SNPs, we inferred common haplotypes within regions of linkage disequilibrium in the genes encoding IGF1, IGFBP-1, and IGFBP-3. There were four blocks of linkage disequilibrium in the gene encoding IGF1; we observed two common (more than 5%) haplotypes in block 1, four in block 2, six in block 3, and six in block 4 (Table 2). IGF1 haplotype frequencies were very similar to those observed in the Caucasian population of the Multiethnic Cohort [37]. The common haplotypes in IGF1 accounted for 95.7 to 99.3% of the chromosomes. The haplotype-tagging SNPs for IGFBP-1 and IGFBP-3 overlapped, resulting in three haplotype blocks covering both genes. We observed five common haplotypes in block 1, four in block 2, and five in block 3 (Table 3). The common haplotypes in genes encoding IGFBP-1/IGFBP-3 accounted for 94.0 to 96.7% of the chromosomes.

The common haplotype patterns in three of the four haplotype blocks in IGF1 were significantly associated with mammographic density (Table 2). There was a modest association between block 1 and mammographic density (*p *= 0.03), and haplotype IGF1-1B (*p *= 0.009) within this block was associated with mammographic density. Block 2 was unrelated to mammographic density. Block 3 was associated with mammographic density in a global test of association (*p *= 0.009) and haplotypes IGF1-3D (*p *= 0.003), IGF1-3E (*p *= 0.02), and IGF1-3F (*p *= 0.005) in this block were each individually associated with mammographic density. For a typical woman in this study (that is, postmenopausal, 57 years old, BMI of 25, no family history of breast cancer, no personal history of benign breast disease, and so on), having two copies of IGF1-3D haplotype is associated with a mammographic density that is 5.9% lower than a woman with zero copies of the haplotype. Block 4 was also associated with mammographic density (*p *= 0.007), and haplotypes IGF1-4D (*p *= 0.02), IGF1-4E (*p *= 0.05), and IGF1-4F (*p *= 0.002) in this block were each individually associated with mammographic density. For the same woman described above, having two copies of the IGF1-4F haplotype is associated with a mammographic density 6.5% lower than a woman with zero copies. Although the study had limited power, no substantial or consistent differences were noted when analyses were stratified by menopausal status (Additional file 1). None of the three haplotype blocks in the genes encoding IGFBP-1/IGFBP-3 were significantly associated with mammographic density in global tests of association (*F *statistic *p *> 0.05; Table 3).

We observed significant associations between four IGF1 haplotype-tagging SNPs and percentage mammographic density. SNP 9 (rs1520220) and SNP 11 (rs2946834) in IGF1 showed the strongest association with mammographic density (Table 4). Those with the G/G genotype for SNP 9 (rs1520220) had a mean percentage mammographic density of 19.6, in contrast with those with the C/C genotype, who had a mean percentage mammographic density of 27.9 (*p *for trend ≤ 0.0001; Table 4). Women with the A/A genotype for SNP 11 (rs2946834) had a mean percentage mammographic density of 23.2, in contrast with those with the G/G genotype, who had a mean percentage mammographic density of 28.2 (*p *for trend = 0.0004; Table 4).

Among the haplotype-tagging SNPs in the genes encoding IGFBP-1/IGFBP-3, SNP 19 (rs4619) (*p *for trend = 0.006; Table 5) was associated with mammographic density. There was an absolute difference of 3.6% between the homozygous variant and homozygous wild-type genotypes (Table 5).

To estimate how often these results might have occurred by chance we conducted permutation testing in which percentage mammographic density was randomly permuted. The most significant association we observed was between SNP 9 (rs1520220) and mammographic density, with a *p *for trend of less than 0.0001. In 10,000 permutations, a *p *value this significant occurred less than 0.05% of the time (multiple comparisons corrected *p *= 0.0005). Age and BMI accounted for 28% of the variation in percentage mammographic density (*R*^2 ^= 0.28); inclusion of SNP 9 in this model explained an additional 1% of variation (*R*^2 ^= 0.29).

In general, the associations between SNPs and haplotypes in IGF1, IGFBP-1, and IGFBP-3 with absolute mammographic density were similar to, but weaker than, those observed with percentage mammographic density. For example, in IGF1 the regression coefficient between haplotype 3D and absolute density was β = -0.31 (95% confidence interval (CI) -0.63 to 0.007), in comparison with β = -0.39 (95% CI -0.65 to -0.14) for percentage mammographic density. The most disparate results were for haplotype 3E, for which β = 0.19 (95% CI -0.15 to 0.53) for absolute density and β = 0.35 (95% CI 0.07 to 0.62) for percentage density. Although we have both genotype information and mammographic density measurements on the cases in this study, we did not believe that the cases could serve as a valid replication set. However, we did conduct analyses including both cases and controls, adjusting for case status, and found that the interpretation of the results remained the same.

## Discussion

This is the first study to comprehensively examine the genetic variation of IGF1, IGFBP-1, and IGFBP-3 in relation to mammographic density. We found strong evidence that common genetic variation in IGF1 is associated with mammographic density. Common haplotype patterns in three out of four haplotype blocks in IGF1; seven specific haplotypes within these blocks were associated with percentage mammographic density. Overall, IGFBP-1/IGFBP-3 haplotype blocks were not associated with mammographic density. We found four SNPs in the gene encoding IGF1 and one SNP in the gene encoding IGFBP-1 that were significantly associated with percentage mammographic density. These results suggest that inherited variation in the IGF pathway has an important role in mammographic density.

We observed absolute mean differences in mammographic density in the range 4 to 8% for specific haplotype-tagging SNPs in IGF1, comparing homozygous variants with homozygous wild-types. These differences are meaningful and are similar to those observed with hormonal interventions. In the Women's Health Initiative Randomized Trial, women randomized to estrogen plus progestin therapy had a mean increase of 6.0% in percentage mammographic density after 1 year, in contrast with 0.9% decrease among women on placebo (*p *< 0.001) [50]. Similarly, the Postmenopausal Estrogen/Progestin Intervention (PEPI) Trial observed significant absolute differences in percentage mammographic density over 1 year ranging from 3.1 to 4.8% for women randomized to different estrogen plus progestin regimens compared with placebo [51]. In a randomized trial of tamoxifen in high-risk women, there was a mean absolute reduction in percentage mammographic density of 5.8%, comparing women on tamoxifen with those on placebo [52]. It is estimated that a difference of 5% in mammographic density is associated with a 7% difference in breast cancer risk [1].

Data from cross-sectional studies suggest that circulating levels of IGF1 are positively associated with mammographic density, whereas levels of IGFBP-3 are inversely associated in premenopausal women [15-17,53]. However, no association between either has been observed in postmenopausal women [15,16,53]. The absence of an association in postmenopausal women may be explained by the difference in levels of circulating IGF1 and IGFBP-3 and mammographic density by menopausal status. Mean levels of IGF1 tend to be higher, and levels of IGFBP-3 tend to be lower in premenopausal women than in postmenopausal women [15,16,53]. Mammographic density is also lower in postmenopausal women than in premenopausal women.

Our ability to detect an association between IGF1 haplotypes with mammographic density in a primarily postmenopausal population may be because genetic variation may more closely reflect the most relevant lifetime exposures of IGF1 (for example tissue level) than single estimates of circulating levels. In addition, genotype is a stable characteristic and is measured with very high accuracy, whereas circulating levels can change over time and are measured with relatively lower accuracy. Thus, measurement error may contribute to the lack of association observed between circulating levels of IGF and postmenopausal mammographic density [15,16]. In addition, circulating levels of IGF are influenced by non-genetic factors such as diet [54,55], physical activity [54,55], exogenous hormones [56-58], and anthropometry [54-56], which may explain a greater portion of variability than genetics.

The variant allele of rs1520220 in IGF1 was inversely associated with mammographic density in the current study. In contrast, the variant allele has been positively associated with circulating levels of IGF1 in premenopausal women in another study [59]. It is unclear what the functional significance of the haplotype-tagging SNPs are. The SNPs in IGF1 associated with mammographic density are located in introns and in the region 3' to the gene, suggesting that if these SNPs are in fact predisposing variants they may function through influencing promotion and/or transcription. It is also possible that these SNPs are in linkage disequilibrium with variants in coding regions, which may directly affect the protein. These associations need to be replicated and it remains to be seen whether the association between IGF1 genetic variation and mammographic density is mediated through circulating levels.

Because of previous evidence linking the -202 polymorphism in the promoter region of IGBP-3 (rs2854744; SNP26) to circulating levels of IGFBP-3 [28,60,61], two studies examined this polymorphism in relation to mammographic density, with inconsistent results [27,28]. One reported a significant association between the variant and mammographic density among premenopausal women (*n *= 206) but not among postmenopausal women (*n *= 206) [28]. The second study reported no association in premenopausal women (*n *= 139) or postmenopausal women (*n *= 153) [27]. We did not observe an association between this polymorphism and mammographic density (*n *= 1,089).

The results of this study suggest that common genetic variation in IGF1 is associated with percentage mammographic density. It is unlikely that these results are due to population stratification, because our study population was 99% Caucasian, and analyses limited to Caucasians did not change the results. To address the issue of false positives arising from multiple comparisons, we conducted permutation testing. On the basis of 10,000 permutations, there is a very low probability that our results are due to chance alone. The magnitude of effects seen in this study is meaningful and suggests that these variants may affect breast cancer risk. However, the same IGF1, IGFBP1, and IGFBP3 genetic variants were assessed with regard to breast cancer in the Multiethnic Cohort (*n *= 1,615 breast cancer cases) and there was no evidence that these common haplotypes were significantly associated with breast cancer risk [62,38]. Additional studies are required to confirm our results with mammographic density and the role of these variants in breast carcinogenesis.

## Conclusion

Common genetic variation in IGF1 is strongly associated with percentage mammographic density.

## Abbreviations

BMI = body mass index; CI = confidence interval; IGF = insulin-like growth factor; IGFBP = insulin-like growth factor binding protein; SNP = single nucleotide polymorphism.

## Competing interests

The authors declare that they have no competing interests.

## Authors' contributions

RMT and DGC were responsible for data analyses, manuscript preparation and editing. PK, SEH, DJH and GAC made substantial contributions to the study design and to the interpretation of data. IC, CAH and MLF were involved in haplotype-tagging SNP selection and contributed substantially to manuscript editing. MNP contributed substantially to manuscript editing. All authors read and approved the final manuscript.

## Supplementary Material

Additional file 1 A Word file containing a table of common IGF1 haplotypes and percentage mammographic density among premenopausal and postmenopausal women, from the Nurses' Health Study. Click here for file

## Figures and Tables

**Table 1:** IGF1, IGFBP-1 and IGFBP-3 haplotype-tagging SNPs

SNP number	SNP ID	Nucleotide change	Minor allele	Allele frequency
IGF1				
Block 1				
1	rs7965399	C/T	C	3.8
2	rs35767	A/G	A	16.2
Block 2				
3	CV11752586	A/G	A	22.5
4	rs1019731	A/C	A	14.6
5	rs2195239	C/G	G	22.7
Block 3				
6	CV346219	A/G	G	28.4
7	rs2373722	A/G	A	8.1
8	rs1549593	G/T	T	14.0
9	rs1520220	C/G	G	19.0
10	CV2801104	G/C	G	14.1
Block 4				
11	rs2946834	G/A	A	33.1
12	rs764876	C/G	C	25.0
13	rs4764695	A/G	G	49.6
14	rs1996656	A/G	G	17.3
IGFBP-1 and IGFBP-3				
Block 1				
15	CV395979	A/G	G	30.2
16	rs1553009	A/G	A	19.7
17	CV395975	G/C	G	24.1
18	rs2201638	A/G	A	2.5
19	rs1065780	G/A	A	38.5
Block 2				
20	rs4988515	C/T	T	4.4
21	rs4619	A/G	G	34.8
22	rs1908751	C/T	T	30.4
23	rs2270628	C/T	T	19.1
Block 3				
24	rs3110697	A/G	A	42.7
25	rs2854746	G/C	C	38.1
26	rs2854744	T/G	T	45.0
27	rs2132570	T/G	T	21.4
Additional SNPs^a^				
28	rs6670	T/A	A	21.1
29	rs2453839	T/C	C	19.8

IGF, insulin-like growth factor; IGFBP, insulin-like growth factor binding protein. ^a^Additional SNPs in IGFBP3 do not fit into any haplotype blocks.

**Table 2:** Common insulin-like growth factor (IGF) haplotypes^a ^and percentage mammographic density, Nurses' Health Study

Block 1 (*p *= 0.03)^b^			Block 2 (*p *= 0.95)^b^			Block 3 (*p *= 0.009)^b^			Block 4 (*p *= 0.007)^b^		
Haplotype	Frequency (percentage)	β^c ^(95% CI)	Haplotype	Frequency (percentage)	β^c ^(95% CI)	Haplotype	Frequency (percentage)	β^c ^(95% CI)	Haplotype	Frequency (percentage)	β^c ^(95% CI)
1A (TG)	82.9	0.16	2A (GCC)	55.0	-0.002	3A (AGGCC)	46.5	0.08	4A (GGAA)	42.4	0.09
		(-0.04 to 0.35)			(-0.15 to 0.14)			(-0.07 to 0.22)			(-0.06 to 0.23)
1B (TA)	**13.3**	**-0.29**	2B (GCG)	22.6	-0.04	3B (AGGCG)	13.6	0.03	4B (ACGA)	14.3	-0.03
		**(-0.52 to -0.07)**^d^			(-0.21 to 0.14)			(-0.18 to 0.24)			(0.24 to 0.19)
Rare	3.9	--	2C (AAC)	12.7	0.03	3C (AGTCC)	11.0	0.15	4C (GGGA)	14.5	0.19
					(-0.17 to 0.24)			(-0.09 to 0.38)			(-0.02 to 0.41)
			2D (ACC)	9.0	0.03	3D (GGGGC)	9.1	**-0.39**	4D (ACGG)	8.8	**-0.34**
					(-0.23 to 0.29)			**(-0.65 to -0.14)**^e^			**(-0.61 to -0.06)**^h^
			Rare	0.8	--	3E (GGGCC)	8.2	**0.35**	4E (GGGG)	**8.1**	**0.28**
								**(0.07 to 0.62)**^f^			**(0.0007 to 0.55)**^i^
						3F (GAGGC)	7.6	**-0.38**	4F (AGAA)	**7.6**	**-0.45**
								**(-0.65 to -0.12)**^g^			**(-0.73 to -0.16)**^j^
						Rare	4.0	--	Rare	4.3	--

CI, confidence interval. ^a^IGF1 haplotype blocks are based on SNPs defined in Table 1. Block 1, SNPs 1 and 2; block 2, SNPs 3 to 5; block 3, SNPs 6 to 10; block 4, SNPs 11 to 14. ^b^Global test of association based on *F *statistic comparing model with haplotypes in block and covariates with covariates only. ^c^β coefficients from models that used square-root-transformed mammographic density as the dependent variable. Multivariate-adjusted models including age (continuous), body mass index (continuous), alcohol consumption (category), age at first birth/parity, history of benign breast disease (yes/no), family history of breast cancer (yes/no), menopausal status/postmenopausal hormone use (premenopausal, postmenopausal never user, postmenopausal current user, postmenopausal past user). Results in bold show significant differences: ^d^*p *= 0.009; ^e^*p *= 0.003; ^f^*p *= 0.02; ^g^*p *= 0.005; ^h^*p *= 0.02; ^i^*p *= 0.05; ^j^*p *= 0.002.

**Table 3:** Common IGFBP-1 and IGFBP-3 haplotypes^a ^and percentage mammographic density, Nurses' Health Study

Block 1 (*p *= 0.17)^b^			Block 2 (*p *= 0.11)^b^			Block 3 (*p *= 0.53)^b^		
Haplotype	Frequency (percentage)	β^c ^(95% CI)	Haplotype	Frequency (percentage)	β^c ^(95% CI)	Haplotype	Frequency (percentage)	β^c ^(95% CI)
1A (AGCGG)	24.7	-0.12	2A (CACC)	35.5	-0.10	3A (GCTG)	37.0	-0.004
		(-0.29 to 0.04)			(-0.25 to 0.06)			(-0.16 to 0.15)
1B (AGGGG)	22.6	-0.06	2B (CCTC)	27.8	-0.07	3B (AGGT)	20.6	-0.005
		(-0.23 to 0.12)			(-0.23 to 0.08)			(-0.19 to 0.17)
1C (AACGA)	19.5	0.07	2C (CGCC)	17.3	0.17	3C (AGGG)	19.4	0.003
		(-0.11 to 0.25)			(-0.03 to 0.37)			(-0.19 to 0.19)
1D (GGCGA)	**18.7**	**0.20**	2D (CGCT)	13.4	0.15	3D (GGGG)	13.0	0.02
		**(0.01 to 0.38)**^d^			(-0.06 to 0.37)			(-0.20 to 0.24)
1E (GGCGG)	10.0	-0.05	Rare	6.0	-0.18	3E (GGTG)	6.7	0.18
		(-0.29 to 0.19)			(-0.57 to 0.20)			(-0.12 to 0.48)
Rare	4.5	-				Rare	3.4	

CI, confidence interval; IGFBP, insulin-like growth factor binding protein. ^a^IGFBP1/IGFBP3 haplotype blocks are based on SNPs defined in Table 1. Block 1, SNPs 15 to 19; block 2, SNPs 20 to 23; block 3, SNPs 24 to 27. ^b^Global test of association based on *F *statistic comparing model with haplotypes in block and covariates with covariates only. ^c^β coefficients from models that used square-root-transformed mammographic density as the dependent variable. Multivariate-adjusted models including age (continuous), body mass index (continuous), alcohol consumption (category), age at first birth/parity, history of benign breast disease (yes/no), family history of breast cancer (yes/no), menopausal status/postmenopausal hormone use (premenopausal, postmenopausal never user, postmenopausal current user, postmenopausal past user). Results in bold show significant differences: ^d^*p *= 0.04.

**Table 4:** Mean percentage mammographic density (MD) according to insulin-like growth factor haplotype-tagging SNPs

SNP	Genotype	*n*	Mean percentage MD^a^	*p *for trend^b^
1	C/C	2	19.1	0.13
	C/T	71	29.5	
	T/T	1,035	26.5	
2	A/A	20	29.1	0.07
	A/G	308	25.0	
	G/G	748	27.4	
3	A/A	51	27.3	0.74
	A/G	388	26.6	
	G/G	662	26.5	
4	A/A	23	25.9	0.89
	A/C	284	26.7	
	C/C	816	26.8	
5	G/G	47	24.7	0.74
	C/G	403	26.6	
	C/C	657	26.8	
6	G/G	88	24.3	**0.05**
	A/G	419	26.4	
	A/A	569	27.6	
7	A/A	7	20.1	**0.007**
	A/G	155	24.7	
	G/G	934	27.4	
8	T/T	20	29.3	0.18
	G/T	255	27.9	
	G/G	776	26.4	
9	G/G	36	19.6	**<0.0001**
	C/G	325	24.8	
	C/C	730	27.9	
10	G/G	19	25.3	0.83
	G/C	262	27.4	
	C/C	809	26.6	
11	A/A	117	23.2	**0.0004**
	G/A	469	26.4	
	G/G	503	28.2	
12	C/C	59	24.3	0.16
	C/G	425	26.5	
	G/G	605	27.2	
13	G/G	270	27.0	0.60
	A/G	570	26.6	
	A/A	278	26.8	
14	G/G	26	26.1	0.82
	A/G	320	26.3	
	A/A	750	26.9	

^a^Multivariate-adjusted models including age (continuous), body mass index (continuous), alcohol consumption (category), age at first birth/parity, history of benign breast disease (yes/no), family history of breast cancer (yes/no), menopausal status/postmenopausal hormone use (premenopausal, postmenopausal never user, postmenopausal current user, postmenopausal past user); ^b^*p *for trend based on inclusion of genotype as an ordinal variable in models with square-root-transformed mammographic density as the dependent variable. Results in bold are significant (*p *≤ 0.05).

**Table 5:** Mean percentage mammographic density (MD) according to IGFBP-1 and IGFBP-3 haplotype-tagging SNPs

SNP	Genotype	*n*	Mean percentage MD^a^	*p *for trend^b^
15	G/G	120	26.4	0.39
	A/G	452	27.3	
	A/A	548	26.2	
16	A/A	36	24.0	0.41
	A/G	377	28.0	
	G/G	707	26.3	
17	G/G	56	25.3	0.75
	C/G	405	26.9	
	C/C	635	26.5	
18	A/A	1	11.9	0.18
	A/G	59	23.8	
	G/G	1,060	26.9	
19	A/A	158	28.4	**0.006**
	G/A	543	27.3	
	G/G	409	24.8	
20	T/T	5	21.7	0.06
	C/T	97	23.6	
	C/C	1,017	27.0	
21	G/G	120	28.2	0.81
	A/G	502	25.6	
	A/A	465	27.3	
22	T/T	108	28.1	0.44
	C/T	441	27.1	
	C/C	557	26.3	
23	T/T	35	28.2	0.67
	C/T	336	26.9	
	C/C	715	26.5	
24	A/A	195	24.8	0.55
	A/G	517	27.4	
	G/G	378	26.5	
25	C/C	148	25.5	0.86
	G/C	511	27.0	
	G/G	383	26.3	
26	T/T	202	26.4	0.87
	T/G	605	26.2	
	G/G	282	26.5	
27	T/T	49	23.4	0.80
	T/G	362	27.4	
	G/G	673	26.4	
28	A/A	47	27.6	0.65
	T/A	359	26.2	
	T/T	700	26.9	
29	C/C	44	24.0	0.16
	T/C	346	26.2	
	T/T	706	27.1	

IGFBP, insulin-like growth factor binding protein. ^a^Multivariate-adjusted models including age (continuous), body mass index (continuous), alcohol consumption (category), age at first birth/parity, history of benign breast disease (yes/no), family history of breast cancer (yes/no), menopausal status/postmenopausal hormone use (premenopausal, postmenopausal never user, postmenopausal current user, postmenopausal past user); ^b^*p *for trend based on inclusion of genotype as an ordinal variable in models with square-root-transformed mammographic density as the dependent variable. The result in bold is significant (*p *≤ 0.05).
