# Supplementary material for: Common genetic variation in IGF1, IGFBP-1, and IGFBP-3 in relation to mammographic density: a cross-sectional study
Source: Breast Cancer Res. 2007 Feb 14;9(1):R18. doi: 10.1186/bcr1655 (PMC1851377; doi:10.1186/bcr1655)
Supplement: Additional file 1 — A Word file containing a table of common IGF1 haplotypes and percentage mammographic density among premenopausal and postmenopausal women, from the Nurses' Health Study. [file bcr1655-S1.doc]

Supplementary Table 1. Common IGF1 Haplotypes (Hap)* and percent mammographic density among premenopausal (Pre) and postmenopausal (Post) women, Nurses’ Health Study

| Block 1 | | | Block 2 | | | Block 3 | | | Block 4 | | |
| --- | --- | --- | --- | --- | --- | --- | --- | --- | --- | --- | --- |
| Hap | Pre | Post | Hap | Pre | Post | Hap | Pre | Post | Hap | Pre | Post |
|  | β (95%CI)^‡^ | β(95%CI) ^§^ |  | β(95%CI)^‡^ | β(95%CI)^§^ |  | β(95%CI)^‡^ | β(95%CI)^§^ |  | β(95%CI)^‡^ | β(95%CI)^§^ |
| 1A | 0.12 | 0.15 | 2A | -0.13 | 0.03 | 3A | -0.21 | 0.15 | 4A | 0.23 | 0.05 |
|  | (-0.37 to 0.60) | (-0.07 to 0.37) |  | (-0.46 to 0.21) | (-0.14 to 0.19) |  | (0.55 to 0.12) | (-0.02 to 0.32) |  | (-0.10 to 0.56) | (-0.11 to 0.21) |
| 1B | -0.34 | -0.27 | 2B | -0.04 | -0.02 | 3B | 0.26 | -0.006 | 4B | 0.11 | -0.05 |
|  | (-0.91 to 0.22) | (-0.51 to -0.03) |  | (-0.46 to 0.38) | (-0.21 to 0.17) |  | (-0.28 to 0.80) | (-0.23 to 0.22) |  | (-0.40 to 0.61) | -0.29 to 0.19) |
|  |  |  | 2C | 0.19 | -0.03 | 3C | 0.60 | 0.01 | 4C | -0.36 | 0.30 |
|  |  |  |  | (-0.28 to 0.66) | (-0.14 to 0.19) |  | (0.07 to 1.12) | (-0.26 to 0.28) |  | (-0.87 to 0.14) | (0.07-0.54) |
|  |  |  | 2D | 0.25 | -0.003 | 3D | -0.09 | -0.49 | 4D | 0.09 | -0.47 |
|  |  |  |  | (-0.43 to 0.93) | (-0.29 to 0.29) |  | (-0.62 to 0.43) | (-0.79 to -0.19) |  | (-0.47 to 0.65) | (-0.79 to -0.15) |
|  |  |  |  |  |  | 3E | 0.27 | 0.38 | 4E | -0.005 | 0.33 |
|  |  |  |  |  |  |  | (-0.51 to 1.06) | (0.08 to 0.68) |  | (-0.77 to 0.76) | (0.03 to 0.63) |
|  |  |  |  |  |  | 3F | -0.40 | -0.38 | 4F | -0.25 | -0.48 |
|  |  |  |  |  |  |  | (-1.04 to 0.24) | (-0.68 to -0.09) |  | (-0.96 to 0.47) | (-0.80 to -0.17) |
|  |  |  |  |  |  |  |  |  |  |  |  |

^*^ IGF1 haplotype blocks based on SNPs defined in Table 1. Block 1: SNPs 1 and 2; Block 2 SNPs 3-5; Block 3 SNPs 6-10; Block 4: SNPs 11-14.

^†^ Global test of association based on F-statistic comparing model with haplotypes in block and covariates to covariates only

^‡^Beta coefficients from models that used square root transformed mammographic density as the dependent variable. Multivariate adjusted models including age (continuous), BMI (continuous), alcohol consumption (category), age at first birth/parity, history of benign breast disease (yes/no), family history of breast cancer (yes/no).

^§^  Multivariate models adjusted for covariates above and PMH use (Never user, current user, past user)
